# High FSH levels impair VEGF secretion of human, frozen-thawed ovarian cortical tissue in vitro

**DOI:** 10.1038/s41598-024-53402-8

**Published:** 2024-02-08

**Authors:** Rebekka Einenkel, Andreas Schallmoser, Nicole Sänger

**Affiliations:** Gynecologic Endocrinology and Reproductive Medicine, University Hospital Bonn, Venusberg-Campus 1, 53127 Bonn, Germany

**Keywords:** Endocrine system and metabolic diseases, Cell biology, Preclinical research

## Abstract

Cryopreservation and reimplantation of human ovarian tissue restore the ovarian hormonal function and fertility due to the preservation of follicles. As the success depends on proper angiogenesis, different approaches aim to support this process. In mice, pretreatment of ovarian tissue with FSH shows increased follicular numbers probably due to the supported angiogenesis by an increased vascular endothelial factor (VEGF) expression. However, in human tissue it remains completely unclear, which effect the hormonal status of the patient has at the time point of reimplantation. Frozen-thawed human ovarian cortical tissue was cultured for 48 h with 0, 1 or 10 ng/mL recombinant human FSH. VEGF-A expression was assessed by ELISA and immunohistofluorescence (IHF) analysis. By IHF, HIF-1α and FSHR expression dependency on culture and FSH concentration was analyzed. Follicles at all stages expressed VEGF-A, which increases during folliculogenesis. Frozen-thawed human ovarian cortical tissue secreted a not statistically different amount of VEGF-A, when cultured in presence of 1 ng/mL FSH (17.5 mIU/mL). However, the presence of 10 ng/mL FSH (175 mIU/mL) significantly decreased VEGF-A expression and secretion. The high FSH concentration increased especially the VEGF-A expression of already growing follicles. The presence of pre-menopausal concentrations of FSH had no significant effect on VEGF-A expression, whereas the presence of elevated FSH levels decreased cortical VEGF-A expression. A hormonal pre-treatment of women with elevated FSH concentrations prior to reimplantation might be considered to support angiogenesis. Here, we show that VEGF-A expression by follicles is affected by FSH dependent on the concentration.

## Introduction

Premature ovarian insufficiency (POI) describes ovarian failure in patients under 40 years old and can be caused by gonadotoxic therapies. As this includes chemotherapy and radiotherapy, oncologic patients are especially affected. Cryopreservation of ovarian tissue can save the follicular pool of patients during gonadotoxic therapy. After reimplantation, the preserved follicles can resume the hormonal function of the ovary, restore the symptoms and provide a source of oocytes in concern of fertility.

Advancements in oncological diagnostic and treatment are continually enhancing the chances of complete healing for patients. Nevertheless, these therapies potentially harm gonads, which varies based on factors such as the specific chemotherapy type, cumulative alkylating agent dose and alkylator equivalent score, and radiation site^1^. The loss of follicles can result in OI.

In fertile women, FSH produced by the anterior pituitary gland is necessary for the maturation of ovarian follicles. FSH stimulates the growth and maturation of follicles in the ovary by receptors on the granulosa cells. Primary follicles already show the expression of FSHR; their dependence on FSH for maturation starts around the establishment of an antrum^2^. The secretion of estradiol and inhibin B by the maturing follicle regulates the release of FSH.

In women with OI, the lack of ovarian response leads the rise of the FSH level. According to the ESHRE Guideline an FSH level above 25 mIU/mL is an indicator for OI.

Since the number of oocyte-containing follicles is finite and lacks self-renewal capabilities, there is no natural restoration of fertility in women. This makes the field of oncofertility so important. It provides patients the opportunity to restore their fertility after therapy. There are several options for fertility preservation in women. Firstly, the administration of gonadotropin-releasing hormone agonist (GnRHa) reduces chemotherapy-induced OI. Secondly, during radiation, surgical transposition can prevent ovarian damage. Thirdly, the cryopreservation of oocytes or ovarian tissue represent additional options. Ovarian tissue cryopreservation (OTC) has become a well-established option in female fertility preservation. Especially in patients, for which hormonal stimulation is no option, ovarian cortical tissue can be cryopreserved. In our cryobank, which is consistent with the report of other cryobanks, breast cancer is the most frequent indication (45% of all OTC patients) followed by lymphomas (22%) and haematological malignancies (8%)^3^. After OTC, the tissue can be stored for years until reimplantation^4^. It is also discussed to delay menopause or replace hormone replacement therapy (HRT) for women with severe perimenopausal symptoms ^5^.

Although appr. 84%^6^–94%^7^ of follicles can be preserved during freezing and thawing, less than 30% of the follicles are expected to survive after reimplantation^8,9^. The fast and effective angiogenesis of the tissue represents one possible issue, which affects follicular reserve. Different approaches now get attention in order to improve reimplantation success. In mouse models, the pre-treatment of the ovarian tissue with FSH during cryopreservation or in situ were proposed^10–12^. There, more follicles survived the grafting after FSH treatment^10,11^ and the number of apoptotic follicles decreased^11^. Moreover, VEGF-A (vascular endothelial factor type A) is increased in the mouse ovarian tissue by FSH treatment^11^. Among other VEGF types, VEGF-A is a key regulator in angiogenesis and most extensively studied. It increases the proliferation and migration of endothelial cells as well as the secretion of proteases for tissue remodeling to support angiogenesis. VEGF-A is spontaneously secreted by human ovarian tissue. However, no study has addressed the effect of FSH on ovarian VEGF expression by human tissue, yet. Moreover, it remains to be elucidated, whether the reimplantation into patients with hypergonadotropic hormonal status due to OI affects the grafting success.

We already showed that frozen-thawed human ovarian cortical tissue needs 48 h to fully recover metabolically^13^. During the first 48 h after thawing, spontaneous VEGF-A secretion by human ovarian cortical tissue increases as well.

The aim of the study was to assess the effect of different FSH concentrations (0, 1 or 10 ng/mL) on VEGF-A expression in frozen-thawed human ovarian tissue.

## Material and methods

All methods were performed in accordance with the relevant guidelines and regulations.

### Ethics approval

This study was conducted in accordance with the relevant guidelines and regulations. The ethical approval for this study was given by the local ethics committee (Ethikkommission an der Medizinischen Fakultät der Rheinischen Friedrich-Wilhelms-Universität Bonn; 007/09). The informed consent was obtained from all patients included in this study before conducting the experiments.

### Ovarian tissue preparation

Ovarian tissue of patients, who underwent fertility preservation counselling and treatment, was used after they gave their written consent to leave a maximum of 10% of the tissue for research (see combined patient information in Table 1). Cryopreservation was performed as described previously^14^. The ovarian tissue has been transported in Custodiol (Dr. Köhler, Germany) at 4 °C after surgical removal. Further processing was performed in Custodiol as well. The tissue was processed under sterile conditions using sterile working benches. To obtain the cortical tissue, medulla has been removed.

**Table 1:** Information of patients included in this study divided according to the experimental methods.

Method	ELISA (see Fig. 2) & IHF (see Fig. 3)	IHF/HE (see Figs. 1, 4, 5)
Number of patients	8	1
Mean age ± SD	29.75 ± 6.12	24
Indication for cryopreservation	6 × Breast cancer, Hodgkin lymphoma, acute lymphocytic leukemia	Hodgkin-lymphoma

*ELISA* enzyme-linked immunosorbent assay, *IHF* immunohistofluorescence, *HE* hematoxylin and eosin staining.

The slow freezing and thawing procedures were performed following our standard operating protocol. Briefly, the tissue was incubated in L-15 Leibovitz´s medium (Life Technologies, USA) containing CryoSure DMSO (10%; WAK Chemie, Germany) and human serum albumin (HSA;10%; Irvine Scientific, USA) for 40 min at 4 °C. The slow freezing process was performed using the IceCube 14S (Sy-Lab, Austria) device. The tissue was frozen to −140 °C in the following regimen: From 4 °C to −6 °C with −1 K/min. Autoseeding started at −6 °C; cooling to −40 °C with −0.3 K/min and to −140 °C at a speed of −1 K/min. The tissue-containing cryotubes (1.8 mL Cryovials; Sarstedt, Germany) were then stored in nitrogen tanks for at least 3 years.

To thaw the tissue samples, vials were firstly incubated at room temperature for 30 s and secondly at 37 °C for 2 min. Then, tissue was exposed to decreasing sucrose (Merck, Germany) solutions (at 0.75, 0.375, 0.187 M) for 15 min each on a shaker at room temperature. Afterwards, the tissue was washed in washing solution containing DPBS CTS (Life technologies, USA) with 10% HSA (Irvine Scientific, USA) for 10 min and for 5 min in a new solution. After thawing, the tissue was used immediately and was either fixated (see “Histology, HE staining and IHF staining”) for the at (after thawing) sample or cultured (see “Tissue Culture”) as described below.

### Tissue culture

Immediately after thawing, ovarian cortical punches of 2 mm diameter and appr. 1 mm of height were cultured at 5% CO_2_, full humidity and 37 °C for 48 h in the presence or absence of FSH as indicated. The used tissue culture medium was based on the ovarian tissue culture medium from McLaughlin et al*.* with minor changes^9^ as used in our previous study^13^. To avoid hormone-like effects of phenol red, McCoy’s medium without phenol red (Cytiva, USA) was used. The medium was supplemented with HEPES (20 mM; AppliChem, Germany), L-glutamine (3 mM; Pan Biotech, Germany) ascorbic acid (50 µg/mL; Sigma-Aldrich, USA), penicillin/streptomycin (LifeTechnologies, UK), insulin, transferrin, selenium (1:200; LifeTechnologies, UK) and HSA (0.1%; Sigma-Aldrich, USA). Recombinant FSH (Bio-Techne, USA) was supplemented to the medium in following concentrations: 0, 1 or 10 ng/mL which corresponded to 0, 17.5 or 175 mIU/mL according to the manufacturer). The use of 1 ng/mL FSH was chosen corresponding to the tissue culture medium of McLaughlin et al.^9^, which was used for follicular growth and maturation.

### ELISA

The incubation medium of the tissue culture was stored until the ELISA was perfomed at -70 °C. The secretion of VEGF-A by the ovarian cortical tissue was assessed using a DuoSet ELISA (catalog number: #DY293B; R&D systems; USA). The ELISA was conducted following the manufacturer's manual. If necessary, the incubation medium was diluted with RnD Reagent, which was provided in the ELISA Kit. The TECAN sunrise (TECAN, Switzerland) was used to assess the absorption at 450 nm with 540 nm as reference wavelength as proposed by the manufacturer. For analysis, an online tool from aatbio was used (Quest Graph™ Four Parameter Logistic (4PL) Curve Calculator." *AAT Bioquest, Inc.*; https://www.aatbio.com/tools/four-parameter-logistic-4pl-curve-regression-online-calculator).

### Histology, HE staining and IHF staining

Tissue fixation, sectioning and staining was performed as previously described ^13^. Tissue punches were fixed directly after thawing or after 48 h of tissue culture in 3.7% formalin in PBS at 4 °C overnight. Fixed fragments were incubated in 15% and subsequently in 30% sucrose in PBS for 10 min. CryoGlue (SLEE; Germany) was used to embed fragments and freeze the samples in liquid nitrogen. The embedded tissue was stored at −20 °C. Tissue sections of 5 µm cut using the MEV cryostat (SLEE; Germany). Sections were dried for 1 h on the slide at 37 °C.

HE staining was performed with a staining kit (Carl Roth; Germany). Therefore, the slides were washed in purified water for 10 s to rinse of the CryoGlue remains and incubated covered with the hematoxylin solution for 6 min. Then, slides were rinsed in tap water once and incubated in hydrochloric acid for 10 s. Blueing was done in tap water for 6 min. Afterwards the sections were covered in eosin and incubated for 1 min. Sections were dehydrated in 90% ethanol (Carl Roth; Germany), absolute ethanol and a second time in absolute ethanol for 5 min. Afterwards, slides were cleared in xylene for an incubation time of 5 min twice. Finally, sections were mounted in Eukitt mounting medium (Sigma-Aldrich; USA).

To prepare for immunohistofluorescence (IHF) staining, antigen retrieval was conducted in antigen retrieval buffer (10 mM Tris base (Sigma-Aldrich; USA), 1 mM EDTA (Sigma-Aldrich; USA), 0.05% Tween 20 (AppliChem; Germany) in *A. dest*, pH = 9.0) for 20 min at 90 °C. The slides were then washed in TBST (20 mM Tris base, 150 mM NaCl (Carl Roth; Germany) and 0.05% TritonX-100 (Sigma-Aldrich; USA) in *A. dest*., pH = 7.6). 5% skim milk powder (Sigma-Aldrich; USA) in TBST was used to block unspecific binding sites for 30 min. The blocking buffer was also used to dilute primary antibodies (PCNA: mouse clone PC10, catalog number: 2586S, CellSignal, UK; VEGF:rabbit polyclonal, catalog number: ab39250, abcam^13^; UK; HIF-1α: mouse clone #241809, catalog number: MAB1536-SP, Bio-Techne, USA; FSHR: rabbit polyclonal, catalog number: 22665-1-AP, Proteintech; USA). The incubation was performed overnight at 4 °C in a hydrated chamber. Staining controls were incubated without primary antibodies. After washing in TBST, sections were incubated with secondary antibodies (Goat anti-Mouse IgG2a Cross-Adsorbed Secondary Antibody, catalog number: A21131, Thermo Fisher Scientific; USA; Goat Anti-Rabbit IgG (H + L), catalog number: E-AB-1055.60, Elabscience; USA) diluted in blocking buffer for 30 min at room temperature. TBST was used to wash slides, which were then rinsed in *A. dest*. before mounting in DAPI-mounting medium (Carl Roth; Germany).

Images were taken using Eclipse Ti2 (Nikon; Japan). Sections stained without primary antibodies were used to adjust settings. To analyze staining intensity of fluorescence images, all images were captured with the same microscope settings. Follicular structures were marked as ROI (region of interest; see Fig. 3B). As the size of the ROI depends on the maturation state, but also where the follicle was cut, the sum of staining intensity was normalized to the area or the ROI. The growing status of the follicles was distinguished by the appearance of the granulosa cells. Primordial cells were defined by a flat layer of granulosa cells. As granulosa cells showed cuboidal shape, the follicles showing monolayer-surrounded oocytes were primary follicles, whereas follicles consisting of multi-layer-surrounded oocytes were secondary follicles. “Growing follicles” describes primary as well as secondary follicles.

### Statistical analysis

Statistical analysis and visualization was performed using Prism 9 (GraphPad Software, USA). Statistical tests were applied as mentioned in figure legends. A *p*-value ≤ 0.05 was considered statistically significant. Effect of FSH treatment was tested with Repeated Measures one-way ANOVA with Dunnett`s or Tukey’s post test. The effect of tissue culture per se was tested using unpaired t test.

### Ethics approval and consent to participate

Patients were informed and gave their written consent. The approval was given by the local ethics committee (Ethikkommission an der Medizinischen Fakultät der Rheinischen Friedrich-Wilhelms-Universität Bonn; 007/09).

## Results

In this study, ovarian cortical tissue was cultured with or without FSH supplementation. To visualize the tissue integrity after thawing and culture, but also the cortical character, HE staining was made.

**Figure 1:**
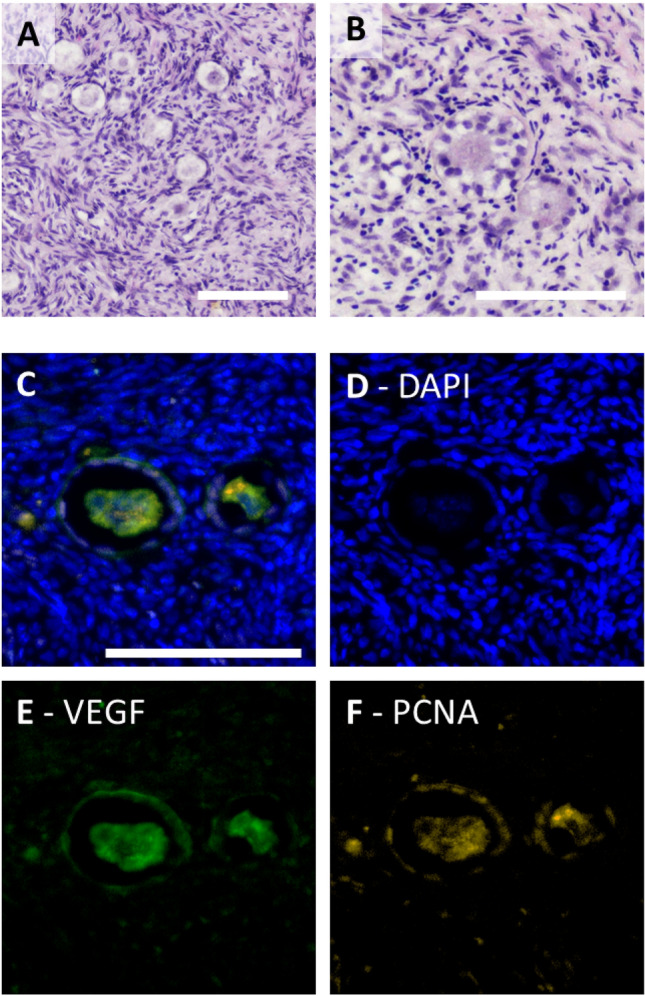
Histological staining of ovarian cortical tissue. Ovarian cortical tissue samples were thawed and cultured with or without FSH supplementation. Tissue punches were fixed and stained with HE (**A, B**) or by immunohistofluorescence (IHF) staining (**C–F**) to show their cortical nature. Representative images are shown for primordial (**A, C–F**) and an early secondary follicle (**B**). White scale bars mark 100 µm.

After thawing as well as after culture, tissue showed well-sustained tissue integrity and distinctive cortical characteristics including primordial as well as primary and secondary follicles (see Fig. 1, 3–5). The PCNA staining additionally confirmed the presence of follicles since PCNA was previously described as suiting follicle marker in the ovarian tissue ^15^.

In order to assess the VEGF-A secretion with or without FSH, the incubation medium after 48 h of tissue culture was used for ELISA measurement.

**Figure 2:**
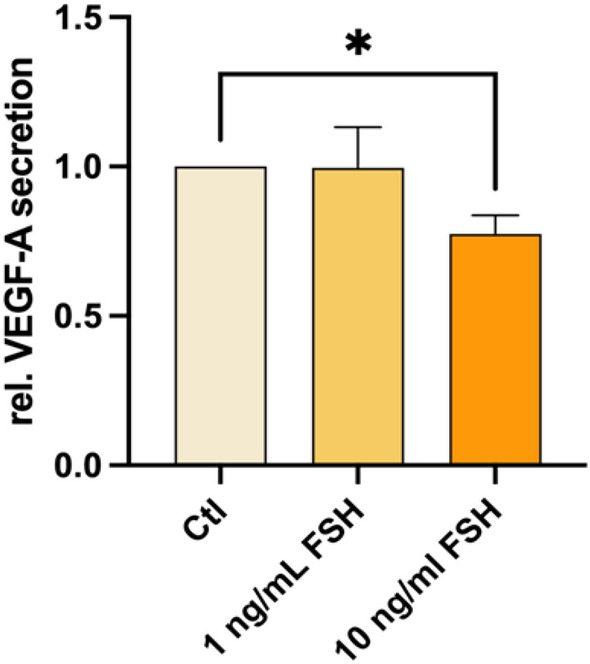
FSH supplementation affects VEGF-A secretion of human ovarian cortical tissue. Tissue punches of thawed ovarian cortex were cultured for 48 h in presence or absence of FSH (1 ng/mL ≙ 17.5 mIU/mL; 10 ng/mL ≙ 175 mIU/mL). VEGF-A secretion was measured by ELISA. Concentrations were normalized to their respective untreated control. Repeated Measures one-way ANOVA with Dunnett’s post test was applied. Bars show mean + SEM. The samples of 8 patients with three biological replicates each were included. *p ≤ 0.05, Ctl—untreated control.

The supplementation of 1 ng/ml FSH did not significantly change the secretion of VEGF-A (see Fig. 2). The addition of 10 ng/mL FSH significantly decreased the VEGF-A release compared to the control (p = 0.016). The mean secretion of 3730 pg/mL was reduced by 10 ng/mL FSH to a mean secretion of 2776 pg/mL. In the samples of all 8 patients a decreasing effect by 10 ng/mL FSH was seen.

To locate VEGF-A signal and confirm the former results, immunohistofluorescence (IHF) images were analyzed. For the image sections of ovarian cortical tissue were stained and quantified.

**Figure 3:**
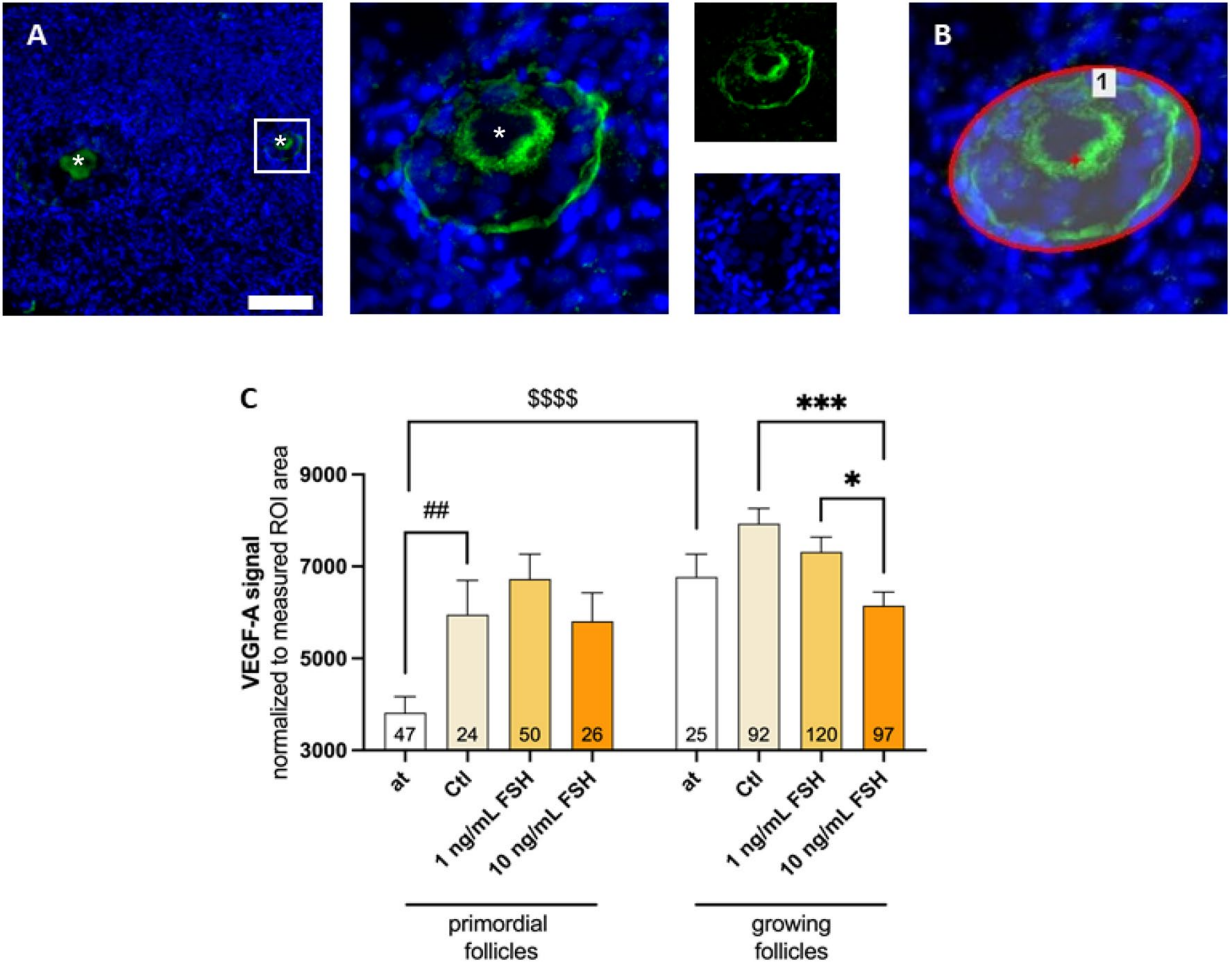
VEGF-A expression by follicles is affected by FSH. Ovarian cortical tissue was either fixated immediately after thawing (at) or cultured in absence (Ctl) or presence of recombinant FSH for 48 h before fixation. Ovarian cortical sections were stained with VEGF-A-specific antibodies and DAPI (nucleus). White asterisks show the location of the oocytes. The white scale bar marks 100 µm (**A**). Follicles were marked as ROI (region of interest; **B**). (**C**) Graphs show the VEGF-A signal as fluorescence intensity normalized to the ROI area in primordial, and growing (early primary to secondary) follicles. Bars show mean + SEM of all visualized follicles in the sections of three patients. In the graph bars, the number of analysed follicles per group is shown. The effect of FSH supplementation was tested by one-Way-ANOVA with Tukey’s Post Test. *p ≤ 0.05, ***p ≤ 0.001. The effect of tissue culture alone comparing “at” with “Ctl” was tested by unpaired t test. ^##^*p* ≤ 0.01 Similarly, the difference between primordial and growing follicles at was assessed by unpaired t test. $$$$ *p* < 0.0001; at—after thawing; Ctl—untreated control.

VEGF-A signals were observed in either close approximation to blood vessels or in the ovarian follicles as described previously^13^. In thawed tissue, growing follicles expressed significantly more VEGF-A compared to primordial follicles (*p* < 0.0001; see Fig. 3).

To examine whether the secretion changes were accomplished by the follicles, the fluorescence intensity of the follicles was analyzed after tissue culture in presence or absence of FSH. During 48 h of culture, VEGF-A expression by follicles increased even without treatment. This was especially significant in primordial follicles (after thawing: 3819 AU; after 48 h of tissue culture: 5953 AU; *p* = 0.0041). Within the primordial follicles, the supplementation of 1 ng/mL FSH led to an increase of VEGF-A (6726 AU) but was not significant. The high concentration of FSH had no significant effect on primordial follicles, but significantly decreased the VEGF-A expression in growing follicles (from 7930 to 6147 AU; *p* = 0.0005).

Since VEGF-A is regulated by several factors with the transcription factor HIF as key regulator, the expression of HIF-1α was assessed by IHF.

**Figure 4:**
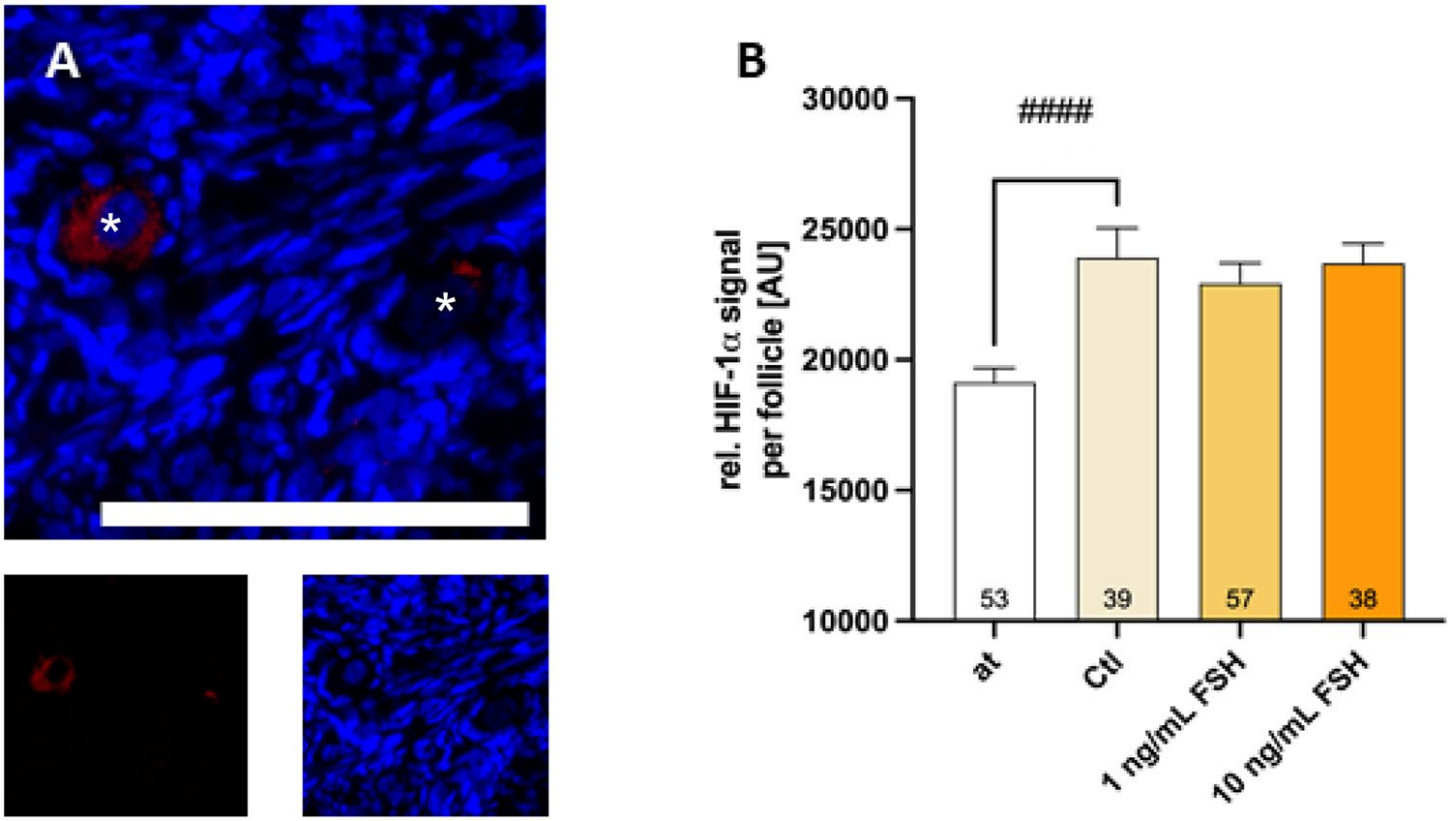
HIF-1α expression is increased during culture and unaffected by FSH supplementation. Human ovarian tissue was either only thawed or additionally cultured for 48 h in tissue culture medium containing different concentrations of FSH. Sections of human ovarian cortex were stained with HIF-1α-specific antibodies and DAPI (nucleus). White bar marks 100 µm (**A**). White asterisks show the location of the oocyte. The sum of HIF-1α fluorescence intensity normalized to the measured follicle area is shown (**B**). Bars show mean + SEM of all found follicles (n = 195; number per group in the bars) in the sections of one patient. The effect of FSH supplementation was tested by one-Way-ANOVA with Tukey’s Post Test. The effect of tissue culture alone comparing “at” with “Ctl” was tested by unpaired t test. ^####^*p* < 0.0001; at—after thawing; Ctl—untreated control.

After thawing (19,124 AU), the tissue culture over 48 h led to a significant increase in the HIF-1α signal (23,904 AU; *p* < 0.0001; see Fig. 4) although the HIF-1α signal was relatively low. The supplementation of FSH did not significantly change HIF-1α signal (22,924 AU and 23,687 AU).

Thus the changes in VEGF-A expression and secretion might be affected and regulated by additional factors others than the transcription factor HIF-1α.

HIF-1α increases the expression of FSHR in granulosa cells^16^. Moreover, FSH treatment in mice leads to a temporary increase of FSH receptor (FSHR) expression^12^. Thus, the expression of FSHR was assessed by IHF.

**Figure 5:**
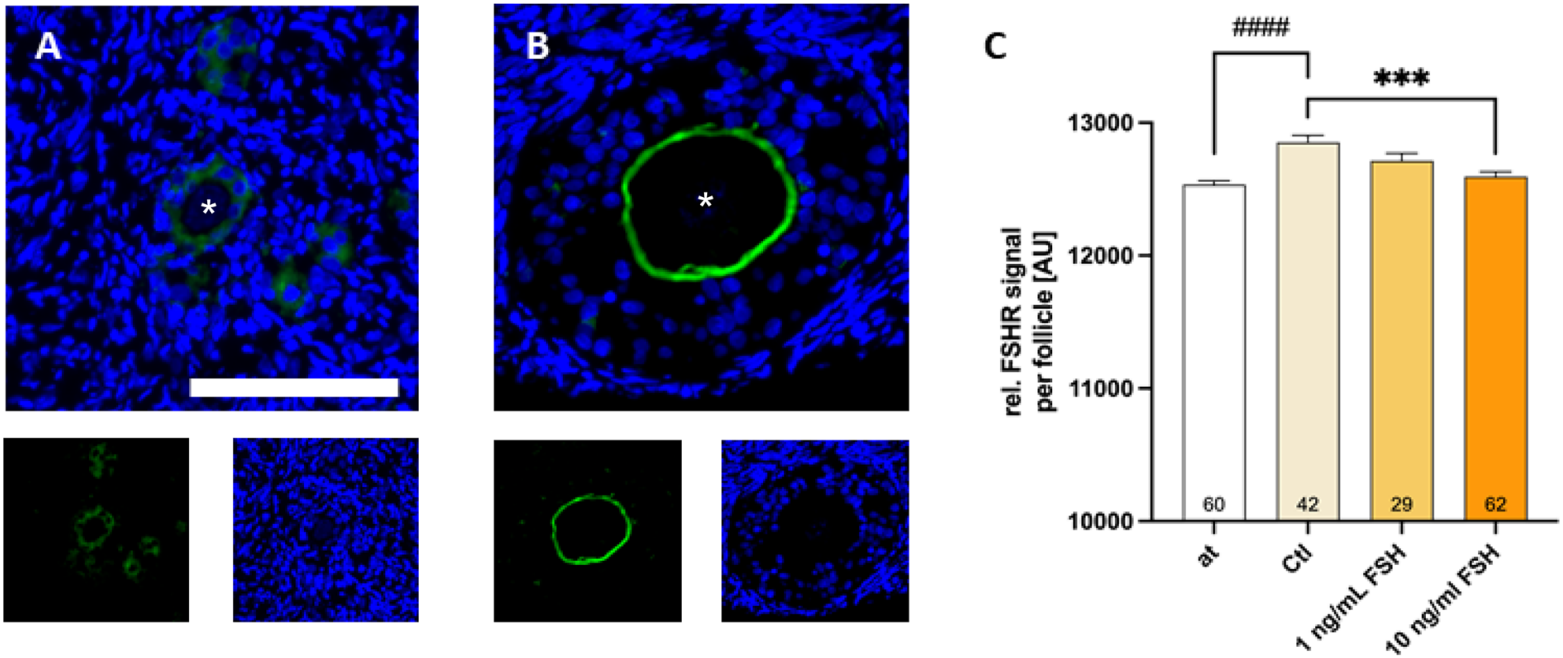
FSH supplementation decreases FSHR expression. Human ovarian tissue was thawed and cultured for 48 h in tissue culture medium with various concentrations of FSH. Either after thawing (at) or after tissue culture, the tissue pieces were fixated. Tissue sections of ovarian cortex were stained with FSHR-specific antibodies and DAPI (nuclear counterstaining). The bar marks 100 µm. Representative images of primary (**A**) and secondary follicles (**B**) are shown. White asterisks show the location of the oocyte. Graph shows the sum of FSHR fluorescence intensity normalized to the measured follicle area (**C**). Bars show mean + SEM of all found follicles (n = 187) in the sections of one patient. The number of follicles in each group can be found in the graph bars. The effect of FSH supplementation was tested by one-Way-ANOVA with Tukey’s Post Test. ***p ≤ 0.001 The effect of tissue culture alone comparing “at” with “Ctl” was tested by unpaired t test. ^####^*p* < 0.0001; at—after thawing; Ctl—untreated control.

Overall follicular FSHR expression was significantly increased by 48 h of tissue culture (12,533 AU to 12,853 AU; *p* < 0.0001; see Fig. 5). With increasing FSH concentration, the FSHR expression decreased. This effect was significant for tissue treated with 10 ng/mL FSH (12,593 AU) compared to the untreated control (12,853 AU; *p* = 0.002). For this analysis, the expression, but not the location was considered. In early follicles, granulosa cells seemed to be slightly positive for FSHR staining intracellularly (see Fig. 5A). Whereas in secondary follicles, the FSHR expression was observed to be located between oocyte and granulosa cells (see Fig. 5B) as shown and described in the literature, where the oocyte as well as the membrane of granulosa cells bordering on the oocyte were positive for FSHR staining in early secondary follicles^17^.

## Discussion

Fresh as well as frozen-thawed ovarian cortical tissue spontaneously secretes VEGF-A^13^. In this study, VEGF-A was shown to be expressed by follicles at all observed growth and maturation levels (primordial to secondary). Besides endothelial structures, especially oocytes, and also granulosa cells expressed VEGF as previously shown by Abir et al*.*^18^. During growth and maturation, the VEGF expression by human follicles rises^19,20^. Similarly, the comparison of primordial follicles to growing follicles showed a significant increase in VEGF-A expression even after normalization to the measured area.

The spontaneously secreted VEGF-A might contribute to the necessary angiogenesis, which has to take place after reimplantation. VEGF-A interacts with VEGFR-1 and VEGFR-2^21^. It acts on various cells, but especially on endothelial cells and induces angiogenesis. Angiogenesis describes the formation of new blood vessels from pre-existing vessels. It is performed in multiple steps and includes the degradation of the basal membrane of the existing capillary, the proliferation of endothelial cells, their migration and the tube formation. Throughout this process, the reimplanted ovarian tissue remains undersupplied. Moreover, the exposition to oxygen after and undersupplied phase, results in the formation of reactive oxygen species (ROS), which are additionally detrimental to the tissue. Thus, it should be a general research aim to understand the mechanisms behind ovarian VEGF secretion and optimization.

Most patients, who perceive OTC, have breast cancer (45% in our cryobank), which can be caused by BRCA1/2 mutations. Various studies showed a correlation between the BRCA status and VEGF expression either in the tumor tissue or in the serum. One the on hand, in the tumor tissue of breast cancer patients, BRCA1/2 mutation carrier showed a significant higher VEGF expression from patients without BRCA mutations^22,23^. On molecular level, it was reported that the BRCA1 interacts with the estrogen receptor alpha, which then inactivates estrogen-induced VEGF transcription in breast cancer cells. Thus, a mutational inactivation of BRCA1 helps to enhance VEGF secretion. On the other hand, in the serum of breast cancer patients with BRCA1 mutation, VEGF is significantly lower than in breast cancer patients without BRCA1 mutation^24^. BRCA1 can enhance HIF-stimulated VEGF transcription; whereas a lack of BRCA1 (due to mutational inactivation) rather decreases VEGF levels^25^. Thus, BRCA and inactivating mutations of BRCA might affect the VEGF secretion, but mechanisms in ovarian tissue are not known yet, but would be of high interest in the context of OTC as well.

This study showed that FSH level affects VEGF-A expression and secretion in frozen-thawed human ovarian cortical tissue. The chosen tissue culture conditions were comparable to those described by McLaughlin et al*.* who started *in-vitro*-growth of follicles in human ovarian tissue under these conditions supplemented with 1 ng/mL FSH^9^. Similarly, human ovarian tissue xenografted to mice which were then treated with LH/FSH lost primordial pool of follicles, because all follicles started to grow and mature^26^. Thus, although FSH-dependency of the follicles starts later during folliculogenesis, FSH has an inducing effect on follicular growth ^9^. After tissue culture supplemented with 1 ng/mL FSH, which corresponds with the concentration used by McLaughlin et al*.*^9^, we would have expected an increase of VEGF expression. In our study, this corresponds to a FSH level of 17.5 mIU/mL, which is comparable to the serum level of pre-menopausal, fertile women. E.g. the ESHRE guideline suggests 25 mIU/mL as cutoff as in peri- and post-menopausal women and women with OI, the FSH levels rise, which is depicted in our study by 10 ng/mL (175 mIU/mL) FSH.

Although there was a slight non-significant increase by 17.5 mIU/mL FSH in primordial follicles analyzed by IHF, we could not show an increase in the secretion measured by ELISA. Luteinized granulosa cells, which strongly express FSHR, react to FSH treatment with an increase in VEGF secretion^27,28^. In mice, FSH administration increases ovarian blood supply mediated by HIF-induced VEGF expression^12,29^. Similarly, rat granulosa cells of antral follicles secrete VEGF after FSH treatment and can induce angiogenesis^30^. VEGF-A expression was slightly increased even in primordial follicles. Thus, indirect effects of the FSH treatment might have led to these changes. Moreover in mice, FSH treatment led to a temporary increase of FSHR expression^12^. After 48 h of tissue culture, follicles expressed significantly more FSHR than before culture. A current study describes HIF-1α to induce FSHR expression in a human granulosa cell line^16^, which was also significantly increased throughout the tissue culture (besides the supplementation of FSH).Throughout the growth and maturation of the follicle FSHR expression increases as well. It is observed that dormant primordial follicles activate growth after reimplantation due to the lack of growth inhibitors. It remains unclear whether the HIF-1α-induced increase in FSHR might also be an expression of follicular growth.

In contrast to the FSH treatment in mice, FSH supplementation to tissue culture decreased overall follicular FSHR expression. This result is similar in post-menopausal women, where a high FSH level is abundant, the FSHR is downregulated by the remaining secondary follicles^31^.

Similar to the VEGF-A expression, tissue culture led to a significant increase of the HIF-1α signal. Although ⌀2 mm biopsy punches of app. 1 mm thick human ovarian cortical tissue were used, limited O_2_ diffusion through the tissue might have caused hypoxic conditions during tissue culture leading to a general rise in HIF-1α. The physiological level of HIF-1α remains unassessed in this study, since the samples after thawing were explanted, transported and processed for several hours under the air O_2_ concentration although cooled. The additional increase in the HIF-1α signal after tissue culture might have caused the rise in VEGF-A as previously shown^13^. So the tissue culture might partly represent the conditions after reimplantation in terms of hypoxia at least in internal part of the tissue. In contrast, the supply of nutrients by the tissue culture medium is ensured as glucose for example diffuse deeper into the tissue than O_2_^32^. This might prevent the tissue from necrotic onset, which would result in harming the tissue and follicles causing their loss after reimplantation. After 48 h of culture, the HIF-1α expression did not differ significantly due to the addition of FSH. In porcine^33^ and murine^34,35^ granulosa cells FSH increases the HIF-1α expression even under hypoxic conditions. These studies used extremely high concentrations of FSH (2 IU/mL; 50 ng/mL), which do not reflect any physiologic condition during reimplantation. Although there might be no effect of FSH on HIF-1α in our study, this does not depict possible changes, which might had happened before. As transcription factors are affected soon after stimulus, changes expression or stability might be not visible anymore after 48 h.

The use of 1 ng/mL (17.5 IU/mL) FSH reflects a physiological serum concentration in pre-menopausal women. In contrast, the higher FSH concentration of 10 ng/mL (175 mIU/mL) used in this study extends the FSH serum concentration of women with diminished ovarian reserve like in post-menopausal women. In contrast to the former discussed effects of FSH, the high concentration led to a significant decrease of VEGF-A expression and secretion. Thus, the reimplantation of ovarian tissue to women with high FSH serum levels might impair angiogenesis of the tissue und the success of restoring ovarian function. In order to support angiogenesis of transplanted ovarian cortical tissue, a hormonal pretreatment similar to HRT could be considered. Appropriately, the grafting in pre-menopausal ovaries is correlated with a more often experienced AMH increased compared to menopausal group^36^. These results led Greve et al*.* to the same proposal of HRT prior to ovarian reimplantation in women with diminished ovarian reserve^36^. More data are required to fully support this strategy.

### Limitations

This is the first paper, where the effects of FSH on the VEGF-A secretion by human frozen-thawed tissue was assessed. Due to the limited access to ovarian tissue and the heterogeneously distributed follicles 8 patients with relatively high follicular counts (mean: 142.9 ± 121.8 SD per 7 mm^2^) were included in this study. Nonetheless, the decreasing effect of 10 ng/mL FSH was not only statistical significant, but also consistent in all 8 patients. The hypothesized increasing effect of 1 ng/mL FSH on the VEGF-A secretion might not be ruled out on molecular level, but was not supported by the data of this study, which might also be caused by the heterogenic nature of the ovarian tissue. Only in primordial follicles, VEGF-A expression was increased, but this change was not significant.

Although a limited sample number was used, the analysis was attempted using the samples of every patient in every treatment group, which reduces the effect of inter-individual differences. This allowed the use of paired statistical tests. Moreover, the same effect was shown with multiple methods.

The ovarian tissue was considered tumor-free by the pathological examination performed routinely after explantation. Although, the included patients and samples cannot considered to be “disease-free”. Moreover, the status of germ line mutations of the included patients are not completely known. Nonetheless, the samples were collected in the context of OTC and thus included the same patient clientele.

The use of as stirred culture might have minimized the oxygen depletion in the tissue due to increased gas exchange. However, the suggested oxygen depletion was probably caused by the limited diffusion into the tissue. Moreover, the expected oxygen conditions after reimplantation are as well limited.

In the IHF, the growing follicles were over-represented, due to their size and the easier identification compared to primordial follicles.

## Conclusion

The regulation of spontaneous VEGF expression by human ovarian cortical tissue is affected by the FSH concentration. The usage of high FSH levels showed a significant impairing effect in vitro. This would compromise the angiogenic capacity. Therefore, the appropriateness of hormone replacement therapy should be evaluated before and during reimplantation in patients with OI.

## Data Availability

The data analysed during the current study are available from the corresponding author on reasonable request.
